# Do Polymorphisms of the TERT, GSTM1, and GSTT1 Genes Increase Laryngeal Cancer Susceptibility in Smokers of Romanian Descent?

**DOI:** 10.3390/medicina58081106

**Published:** 2022-08-16

**Authors:** Corina Iulia Cornean, Andreea Catana, Alma Aurelia Maniu

**Affiliations:** 1 Department of Otolaryngology-Head and Neck Surgery, Iuliu Hatieganu University of Medicine and Pharmacy, 400006 Cluj-Napoca, Romania; 2 Department of Molecular Sciences, Iuliu Haţieganu University of Medicine and Pharmacy, 400349 Cluj-Napoca, Romania

**Keywords:** laryngeal cancer, TERT, genetic susceptibility, glutathione S-transferases, single-nucleotide polymorphism

## Abstract

*Background and Objectives*: The aim of this study was to investigate the association between smoking status and single-nucleotide polymorphism in candidate genes that had a known association with smoking-related tumors in previous studies and to explore their link to laryngeal cancer risk in a population of northern Romanian descent. The genes selected have key functions in xenobiotic metabolism (GSTs: the glutathione S-transferases family: GSTM1 and GSTT1) and chromosomal management (TERT). *Materials and Methods*: The genotype frequencies of TERT_Rs2736100_ and the GST subfamilies (GSTM1 and GSTT1) were determined using the polymerase chain reaction-restriction fragment length polymorphism (PCR-RFLP) technique. The relationship between the polymorphisms and the risk of laryngeal cancer was analyzed in a retrospective case–control study of 92 laryngeal cancer cases and 101 controls, all of whom were smokers. *Results*: Subjects presenting the GSTT1-null variant had a two-fold increase in risk (OR = 2.05, 95% CI = 1.07–3.95, *p* = 0.02). While no individual risk was observed for the TERT_Rs2736100_ polymorphism, stratification based on gender revealed a nine-fold increase in risk for carriers of the “C” allele in the heterozygote variant who were male (OR = 9, 65% CI = 3.51–26.51, *p* = 0.0000). *Conclusions*: The results showed that the GSTT1-null genotype and the mutant heterozygote variant of TERT_Rs2736100_ genes may play a significant role in laryngeal cancer susceptibility in subjects of northern Romanian descent. There may be no association between the susceptibility to laryngeal carcinoma and the GSTM1 polymorphism. The results could not confirm the carcinogenic influence smoking has on laryngeal cancer development for the studied polymorphisms.

## 1. Introduction

To date, carcinogenesis has been linked with genetic and environmental/lifestyle factors. However, the multitude of possible combinations between them has made the identification of the exact etiology a continuous struggle [1,2]. Scholarly research, performed on various populations throughout the years, has identified differences in the occurrence rate of malignant tumors when exposed to environmental risk factors. These findings highlight the importance of the innate genetic background of each individual; exposure alone does not invariably lead to cancer [3,4]. Among head and neck cancers, laryngeal cancer has a relatively high incidence rate and is mostly found in males within Central and Southern Europe, with a steady yearly increase rate [5].

Epidemiological studies focusing on the development of this malignancy have identified a wide range of influential factors [6,7], among which cigarette smoking poses a major carcinogenic risk factor considering that most patients (close to 80%) have a history of tobacco consumption [8]. In such a study, Ramroth et al. [9] reported an increased incidence rate of laryngeal cancer in both smokers and non-smokers exposed to a smoking environment compared to those in a non-smoking environment.

Concurrently, the aforementioned risk tends to vary greatly depending on the number of cigarettes consumed, the duration of smoking, and the tumor subsites [9,10,11]. In studies on laryngeal cancer, Guoxiang et al. [12] observed that cigarette consumption of more than 20 cigarettes per day for at least five years significantly increased the incidence rate of this pathology, while Pantel et al. [8] reported a minimum smoking period of 31–34 years until the onset of laryngeal cancer.

The characterization of laryngeal cancer through genetic testing over the years has led to the identification of several candidate genes with key functions linked to the carcinogenic process in populations around the world [13]. However, for populations of Romanian descent, this form of genetic association with laryngeal cancer remains unknown. The purpose of this study was to investigate, within this ethnic group, the association between smoking status and candidate genes with roles in chromosomal maintenance (TERT: telomerase reverse transcriptase) and the carcinogenic metabolization of toxins (GSTs: the glutathione S-transferases family: GSTM1, GSTT1), and to explore their role in laryngeal squamous cell carcinoma (LSCC) development.

## 2. Materials and Methods

In this study, we performed a retrospective case–control study on 92 LSCC subjects and 101 controls, all smokers, from the Department of Genetics and the Department of Molecular Sciences, “Iuliu Haţieganu” University of Medicine and Pharmacy, Cluj-Napoca, Cluj County, Romania. Details regarding the age, gender, and smoking duration (in years) of the subjects were recorded. Grouping according to yearly consumption was carried out according to the mean value: light smokers (<29 years) and heavy smokers (≥29 years).

### DNA Extraction and Genotyping

The polymerase chain reaction-restriction fragment length polymorphism (PCR-RFLP) technique was used to identify the genotype frequencies of the three candidate polymorphisms. DNA was extracted from ethylenediaminetetraacetic acid (EDTA) blood using specific DNA extraction kits and put through PCR fragment amplification and successive thermocycling conditions (Mastercycler Gradient, Eppendorf AG, Hamburg, Germany) according to the manufacturer’s protocol (Table 1).

## 3. Statistical Analysis

The chi-square test and Fisher’s exact test were performed to analyze the established categorical data sets (demographic characteristics, tobacco consumption, and genotypes). Logistic regression models were used to identify possible associations between individual genotypes and outcome parameters. The analysis was initially carried out using the polymorphism of each gene as a predictor variable and subsequently in combination with other predictor variables. All genotypes were converted into dummy variables for this process. Statistical analysis was carried out using the Epiinfo (version 7.2.3.1) software. Crude and adjusted odds ratios (OR) with corresponding 95% confidence intervals (CIs) were calculated to estimate the risk and strength of association. The homozygote wild-type genotype of each polymorphism was used as the reference category for comparative analysis. Results were considered significant if the value of probability (*p*) did not exceed 0.05. OR and CI were not calculated when data were too sparse. The final results were then subjected to false discovery rate verification.

## 4. Results

The aforementioned study population was divided according to age, gender, and tobacco consumption, as reported in Table 2. Though the majority of subjects were males, the difference was not considered statistically significant (*p* = 0.41). The predominant age group consisted of subjects over 60 years of age without a significant difference between cases and controls (*p* = 0.06). Among the smoking population, the most common group consisted of moderate smokers, but the difference was not statistically significant (*p* = 0.14).

The identified genotypes and allele frequencies of TERT_Rs2736100_, GSTM1, and GSTT1 are reported in Table 3. The genotype distribution of each polymorphism in the control group was in keeping with the expected values of the Hardy–Weinberg equilibrium (data not shown) after parameter calculations using Internet-based programs [14]. For TERT_Rs2736100_, the mutant homozygote variant CC was the most common (39.13%) in cases, while the mutant heterozygote variant AC appeared more frequently in controls (42.57%). Though the GSTM1-null genotype was more common in laryngeal cancer subjects than in controls (55. 43% vs. 48.51%), as opposed to the GSTM1-positive genotype, which showed a reverse distribution (51.49% vs. 44.57%), the results were not statistically significant (*p* = 0.38). The GSTT1-positive genotype was more frequent in controls (80.20%) than in cases (66.30%), and the difference was statistically significant (*p* = 0.03). As to the concurrent GSTM1/GSTT1 genotypes, the dual positive genotype had the highest frequency among controls (42.57%), while the GSTM1-null/GSTT1-positive genotype had the second-highest distribution among both cases and controls. However, no statistically significant difference in the genotype and allele distribution was identified.

The comparative analysis of genetic models for the variant carriers of laryngeal cancer risk is shown in Table 4. The studied polymorphisms were initially treated as independent risk factors in a logistic regression model before further covariates were added to the existing model. No significant association was observed between laryngeal cancer risk and TERT_Rs2736100_, GSTM1, and concurrent GSTM1/GSTT1 genotypes in any of the studied genetic models. However, GSTT1-null showed a two-fold increase as a protective cancer factor when compared to the reference genotype (OR = 2.05, 95% CI = 1.07–3.95, *p* = 0.02).

Table 5 and Table 6 show the results of the analysis after subject stratification according to genotype and secondary variables such as age (in years), gender, and smoking status (in years). The stratification based on gender identified male subjects possessing the AC genotype of Rs2736100 as a high-risk group in terms of laryngeal cancer susceptibility, with an almost nine-fold risk (OR = 9.65, 95%CI = 3.51–26.51, *p* = 0.00000) compared to the reference category (Table 5). No other statistically significant association was identified for the genotypes of Rs2736100. Similar stratification performed on GSTM1, GSTT1 (data not shown), and the combined GSTM1/GSTT1 (Table 6) genotypes did not reveal any statistical significance when compared to the reference genotypes.

## 5. Discussion

This study is the first to investigate the polymorphisms of TERT_Rs2736100_ and the GST supergene family (GSTM1, GSTT1) as potential risk factors for laryngeal cancer among smokers of northern Romanian descent. Studies on the existing relationships between genetic polymorphisms and cancer development have been extensive, although the findings are yet to be fully conclusive. The applicability of TERT_Rs2736100_ has been well documented in connection to the neoplastic process [15,16] due to its essential involvement in telomere maintenance and regeneration [17,18]. Under normal circumstances, telomerase activity shows strong regulation in somatic cells, while reactivations have been found in up to 80–90% of tumor cells [19]. Meta-analyses have shown that the predominant neoplastic association occurs in lung cancer [20,21], while other studies refer to pathologies such as glioma [22] and myeloproliferative neoplasms [23].

Among the studied population, the results of our research could not find any positive association between TERT_Rs2736100_ and laryngeal cancer as an independent risk factor for any of the individual genotypes. However, subsequent stratification based on gender highlighted the association between laryngeal cancer and the mutant heterozygote genotype AC. Males carrying at least the “C” allele were at far higher risk for LSCC than those without (OR = 9.65, 95% CI = 3.51–26.51, *p* = 0.0000). This result confirms the importance of gender distribution in neoplastic development [5]. In assessing the individual alleles of RS2736100 as influencing factors in cancer development, Snetselaar et al. [18] performed a meta-analysis and found that the “C” allele of Rs2736100 was positively associated with cancer (pooled OR = 1.16, 95% CI = 1.09–1.23) and negatively associated with non-cancerous diseases (pooled OR = 0.81, 95% = CI 0.65–0.99). These results tend to corroborate the influence that the “C” allele may have on laryngeal cancer development, though further research on these subjects is required. Analysis based on smoking status showed no positive association between TERT_Rs2736100_ and laryngeal cancer susceptibility, contrary to those studies reporting that a minimum period of smoking ranging from 5 to 30 years caused a high neoplastic risk and that tobacco smoke was a stable and independent major risk factor [8,12,24,25].

Due to their fundamental role in the detoxification process of carcinogenic reactive metabolites, the literature has characterized GSTM1 (class Mu) and GSTT1 (class Theta)—of the GST supergene family—as carcinogen-metabolizing genes [26]. In head and neck cancers (HNC) especially, this process has specific relevance for tobacco consumption [27,28,29]. Meta-analyses, such as those performed by Zhang et al. [30], Liu et al. [31], and Ying et al. [32], have reported on the significance of function loss through deletion as an individual neoplastic risk factor for members of the GST superfamily, and GSTM1-null in particular. Results regarding the GSTT1-null genotype, however, appear more varied. In their studies, Li et al. [33] found no such association, Unal et al. [34] reported that GSTT1-null significantly increased in laryngeal cancer patients when compared with the non-smoking controls (*p* = 0.04), and Lourenço et al. [35] noted that both the GSTM1 and GSTT1 pathways are important determinants of head and neck squamous cell carcinoma in smokers.

The results of the present study could not corroborate this particularity of the GSTM1 gene for our Romanian population group. However, our analysis of the GSTT1-null genotype highlights the implications that deletion has concerning its influence on cancer progression within this pathology. When applying the same stratification based on smoking status, we could not validate the notion that tobacco consumption appears to boost individual sensitivity as patients with reduced detoxification cannot operate on the same level in terms of expelling the toxic compounds which characterize tobacco intake [36]. In opposition, Tian et al. [37] found a positive synergic effect between the GSTT1-null genotype and heavy smoking during the carcinogenesis of LSCC (OR = 3.51, 95% CI 2.05–5.01; OR = 2.99, 95% CI 2.00–4.49). Peter et al. [38] also reported that the GSTM1-null genotype was associated with an increased risk for HNSCC (OR = 1.3, 95% CI 1.0–1.6) while also observing a higher risk in heavy smokers (OR = 4.2, 95% CI 1.6–4.3) with deletion compared to subjects without (OR = 2.6, 95% CI 1.6–4.3). Subsequent investigation of the concurrent GSTM1-GSTT1 genotypes within our population could not confirm the hypothesis that the presence of at least one deleted genotype within the GST supergene family in individuals can increase neoplastic development. In a similar examination of both the GSTM1/GSTT1 genotypes, Acar et al. [39] noted that light-to-medium smokers with both GSTs-null genotypes had the highest risk for supraglottic LSCC in the early period.

Because this is the first study to include the examination of these specific genes in a Romanian population group, we consider our results as preliminary to further independent studies required to verify our results with a larger sample size/geographic distribution within the territory of Romania.

## 6. Conclusions

Our results highlight the importance of deletion in GSTT1 as an independent risk factor for neoplastic development in laryngeal cancer. Carriers of the TERT Rs2736100 mutant heterozygote genotype (AC) have a significantly higher risk of laryngeal cancer compared to those with the normal wild-type genotype. There may be no association between the susceptibility to laryngeal carcinoma and the GSTM1-null polymorphism in a population of Romanian descent. Although smoking has been deemed a major carcinogenic risk factor in laryngeal cancer development, no such relationship could be confirmed within this ethnic group for the studied polymorphisms.

## Figures and Tables

**Table 1:** Genotyping technique.

Polymorphism	DNA Extraction Kit	Specific Primer Sequence^ *a*^	Temperature	Time	Cycles
TERT_Rs2736100_	Wizard Genomic DNA Purification Kit	GAAAAGCAGGGCGGGGGCACAAGCTA [A/C] AGAAACACTCAACACGGAAAACAAT	95 °C	10 min	X1
			92 °C	15 s	X40
			60 °C	1 min	
GSTM1	TaqMan™ Genotyping Master Mix	PF 5′-GAACTCCCTGAAAAGCTAAAGC-3′	94 °C	5 min	X1
		PR 5′-TTCCTTACTGGTCCTCACATCTC-3′	94 °C	1 min	X35
GSTT1		PF 5′-TTCCTTACTGGTCCTCACATCTC-3′	58 °C	1 min	
		PR 5′-TCACCGGATCATGGCCAGCA-3′	72 °C	1 min	
			72 °C	10 min	Final extension

*^a^*: Abbreviations: forward primer (PF); reverse primer (PR); A: adenine; G: guanine; C: cytosine; T: thymine.

**Table 2:** Distribution of patients and controls according to demographic characteristics.

Variables	Cases *n* = 92 (%)	Controls *n* = 101 (%)	χ^2 *a*^	*p*-Value
Gender				
Male	87 (94.57%)	92 (91.09%)	0.86	0.41
Female	5 (5.43%)	9 (8.91%)		
Age (years)				
Mean	60 ± 7.79	62 ± 6.59	3.85	0.06
<62	54 (58.7%)	45 (44.44%)		
≥62	38 (41.30%)	56 (55.45%)		
Tobacco consumption *^b^*				
Mean	29 ± 7.92	28 ± 7.21		
<29 years	31 (33.70%)	45 (44.55%)	2.37	0.14
≥29 years	61 (66.30%)	56 (55.45%)		

*^a^* Two-sided chi-square test (χ^2^). *^b^* Tobacco consumption: light smoking (<29 years), heavy smoking (≥29 years).

**Table 3:** Genotype and frequency distribution of TERT_Rs2736100_, GSTM1, and GSTT1 genotypes in subjects and controls.

Polymorphism	Cases *n* = 92 (%)	Controls *n* = 101 (%)	χ^2 *a*^	*p*-Value *^e^*
**TERT_Rs2736100_** *^b^*				
AA	24 (26.09%)	26 (25.74%)	1.51	0.46
AC	32 (34.78%)	43 (42.57%)		
CC	36 (39.13%)	32 (31.68%)		
Alele C	104 (%)	107 (%)	0.49	0.53
Alele A	80 (%)	95 (7%)		
**GSTM1(+/−)** *^c^*				
positive (+)	41 (44.57%)	52 (51.49%)	0.92	0.38
null (−)	51 (55.43%)	49 (48.51%)		
**GSTT1(+/−)** *^c^*				
positive (+)	61 (66.30%)	81 (80.20%)	4.78	0.03
null (−)	31 (33.70%)	20 (19.80%)		
**GSTM1/GSTT1** ^*d*^				
Genotype 00 [GSTM1(+)/GSTT1(+)]	28 (30.43%)	43 (42.57%)	5.67	0.12
Genotype 01 [GSTM1(+)/GSTT1(−)]	12 (13.04%)	9 (8.91%)		
Genotype 10 [GSTM1(−)/GSTT1(+)]	33(35.87%)	38 (37.62%)		
Genotype 11 [GSTM1(−)/GSTT1(−)]	19 (20.65%)	11 (10.89%)		

*^a^* Two-sided chi-square test (χ^2^). *^b^* TERT genotypes: homozygote wild-type (AA); mutant heterozygote (AC); mutant homozygote (CC). *^c^* GSTM1 genotypes: GSTM1(−), null genotype (deletion); GSTM1(+), positive genotype (no deletion); GSTT1 genotypes: GSTT1(−), null genotype (deletion); GSTT1(+), positive genotype (no deletion). *^d^* Concurrent GSTM1/GSTT1 genotypes: wild-type genotype [GSTM1(+)/GSTT1 (+)], noted genotype 00; positive GSTM1-null GSTT1[GSTM1(+)/GSTT1(−)], noted genotype 01; null GSTM1-positive GSTT1[GSTM1(−)/GSTT1(+)], noted genotype 10; double deletion [GSTM1(−)/GSTT1(−), noted genotype 11]. *^e^* Bold values indicate statistical significance per Fisher’s exact test after application of false discovery rate (FDR) correction, *p* < 0.05.

**Table 4:** Relationship between TERT_Rs2736100_, GSTM1, and GSTT1 genotypes, and laryngeal cancer susceptibility.

Genetic Model	Crude OR *^a^* (95% CI)	*p*-Value	Adjusted OR *^d^* (95% CI)	*p*-Value ^*d*^
**TERT_Rs2736100_**				
AA	(Ref) 1.00 *^b^*		(Ref) 1.00 *^b^*	
AC	0.80 (0.36–1.76)	0.58	0.80 (0.39–1.65)	0.55
CC	1.21 (0.55–2.70)	0.7	1.21 (0.58–2.53)	0.59
Dominant *^b^*	0.98 (0.49–1.97)	1	0.98 (0.51–1.87)	0.95
Recessive *^b^*	1.38 (0.73–2.61)	0.29	1.38 (0.76–2.50)	0.27
Allele C	(Ref) 1.00 *^b^*	0.53	(Ref) 1.00 *^b^*	0.48
Allele A	1.15 (0.75–1.75)		1.15 (0.77–1.72)	
**GSTM1(+/−)**				
GSTM1(+)	(Ref) 1.00 *^c^*		(Ref) 1.00 *^c^*	
GSTM1(−)	1.31 (0.72–2.42)	0.38	1.34 (0.76–2.36)	0.3
**GSTT1(+/−)**				
GSTT1(+)	(Ref) 1.00 *^c^*		(Ref) 1.00 *^c^*	
GSTT1(−)	2.05 (1.02–4.19)	0.03	2.05 (1.07–3.95)	**0.02** ^*e*^
**GSTM1/GSTT1**				
GSTM1(+)/GSTT1(+)	(Ref) 1.00 *^c^*		(Ref) 1.00 *^c^*	
GSTM1(+)/GSTT1(−)	2.03 (0.68–6.25)	0.21	2.04 (0.76–5.49)	0.15
GSTM1(−)/GSTT1(+)	1.33 (0.64–2.74)	0.49	1.33 (0.68–2.59)	0.39
GSTM1(−)/GSTT1(−)	2.62 (1.01–7.12)	0.03	2.65 (1.09–6.40)	0.02

*^a^* OR: odds ratio; 95% CI: 95% confidence interval. *^b^* TERT genotypes: dominant ((AC + CC) vs. AA), recessive (CC vs. (AA + AC)). *^c^* Reference categories (OR = 1): the wild-type genotype and allele for each individual polymorphism. *^d^* Adjusted OR, calculated in a logistic regression model without control for gender, age, or tobacco consumption. *^e^* Bold values indicate statistical significance after application of FDR correction, *p* < 0.05.

**Table 5:** Stratification analysis of the association between TERT_Rs2736100_ and laryngeal cancer.

TERT_Rs2736100_ C/A							
Variables	Number of Cases/Controls			Adjusted OR *^a^*^,*b*^ (95% CI)			
	AA *^c^*	AC *^c^*	CC *^c^*	AC vs. AA *^c^*	CC vs. AA *^c^*	(AC + CC) vs. AA *^c^*	CC vs. (AA + AC) *^c^*
Gender							
Male	23/24	31/37	33/31	**9.65 (3.51–26.51), *p* = 0.0000** *^d^*	1.11 (0.52–2.35), *p* = 0.78	0.82 (0.42–1.62), *p* = 0.58	1.27 (0.68–2.35), *p* = 0.44
Female	1/2	1/6	3/1	0.33 (0.01–8.15), *p* = 0.5	5.99 (0.22–162.17), *p* = 0.26	1.14 (0.07–16.91), *p* = 0.92	12 (0.77–186.36), *p* = 0.05
Age (in years)							
<62	16/13	17/16	21/16	0.86 (0.31–2.34), *p* = 0.77	1.07 (0.40–2.88), *p* = 0.87	0.96 (0.40–2.30), *p* = 0.93	1.27 (0.56–2.86), *p* = 0.55
≥62	8/13	15/27	15/16	0.90 (0.30–2.66), *p* = 0.85	1.52 (0.49–4.70), *p* = 0.46	1.47 (0.53–4.05), *p* = 0.44	1.63 (0.68–3.89), *p* = 0.27
Smoking status (in years)							
<29	7/12	8/17	16/16	0.80 (0.23–2.82), *p* = 0.73	1.71 (0.53–5.47), *p* = 0.35	1.24 (0.42–3.63), *p* = 0.68	1.933 (0.76–4.91), *p* = 0.16
*p* ≥ 29	17/14	24/26	20/16	0.76 (0.30–1.86), *p* = 0.54	1.02 (0.39–2.70), *p* = 0.95	0.90 (0.39–2.07), *p* = 0.81	1.21 (0.55–2.68), *p* = 0.62

*^a^* Abbreviations: OR: odds ratio; 95% CI: 95% confidence interval. *^b^* Adjusted OR for covariates such as gender, age (in years), and tobacco consumption (in years) in a logistic regression model for each stratum. *^c^* TERT genotypes: homozygote wild-type (AA); mutant heterozygote (AC); mutant homozygote (CC); dominant ((AC + CC) vs. AA), recessive (CC vs. (AA + AC)). *^d^* Bold values indicate statistical significance after application of FDR correction, *p* < 0.05.

**Table 6:** Stratification analysis of the association between the concurrent GSTM1/GSTT1 genotypes and laryngeal cancer.

Combined GSTM1/GSTT1							
Variables	Number of Cases/Controls				Adjusted OR *^a^*^,*b*^ (95% CI)		
	Genotype 00 ^*c*^	Genotype 01 ^*c*^	Genotype 10 ^*c*^	Genotype 11 ^*c*^	Genotype 01 vs. Genotype 00	Genotype 10 vs. Genotype 00	Genotype 11 vs. Genotype 00
Gender							
Male	26/40	11/7	32/34	18/11	2.41 (0.83–7.03), *p* = 0.10	**1.44 (0.72, 2.88), *p* = 0.29** *^e^*	**2.51 (1.02, 6.17), *p* = 0.04** *^e^*
Female	2/3	1/2	1/4	1/0	0.75 (0.03–14.99), *p* = 0.84	0.37 (0.03–17.50) *p* = 0.85	*^d^*
Age (in years)							
<62	15/17	8/4	22/20	9/4	2.26 (0.56–9.06), *p* = 0.23	1.24 (0.49–3.13), *p* = 0.63	2.73 (0.69–10.78), *p* = 0.13
≥62	13/26	4/5	11/18	10/7	1.60 (0.36–6.98), *p* = 0.53	1.22 (0.44–3.33), *p* = 0.69	2.85 (0.88–9.23), *p* = 0.07
Smoking status (in years)							
<29	8/23	6/4	16/14	1/4	2.87 (0.57–14.27), *p* = 0.19	3.28 (1.11–9.64), *p* = 0.02	0.71 (0.06–7.41), *p* = 0.77
≥29	20/20	6/5	17/24	18/7	1.19 (0.31–4.57), *p* = 0.78	2.57 (0.88–7.49), *p* = 0.07	0.70 (0.29–1.70), *p* = 0.44

*^a^* Abbreviations: OR: odds ratio; 95% CI: 95% confidence interval. *^b^* Adjusted OR for covariates such as gender, age (in years), and tobacco consumption (in years) in a logistic regression model for each stratum. *^c^* Concurrent GSTM1/GSTT1 genotypes: reference genotype noted genotype 00; positive GSTM1/null GSTT1 noted genotype 01; null GSTM1/positive GSTT1 noted genotype 10; double deletion noted genotype 11. *^d^* OR could not be calculated due to zero values in one category. *^e^* Bold values indicate statistical significance after application of FDR correction, *p* < 0.05.

## Data Availability

The data presented in this study are available on request from the corresponding author. The data are not publicly available due to the research being performed on a retrospective database.

## Abbreviations

TERT	telomerase reverse transcriptase
GST	glutathione S-transferases
GSTM1	glutathione S-transferase class Mu
GSTT1	glutathione S-transferase class Theta
LSCC	laryngeal squamous cell carcinoma
PF	forward primer
PR	reverse primer
HNC	head and neck cancer
SNP	single-nucleotide polymorphism
ETDA	ethylenediaminetetraacetic acid
PCR–RFLP	polymerase chain reaction-restriction fragment length polymorphism
HWE	Hardy–Weinberg equilibrium theory
A	adenine
C	cytosine
GST null genotype (-)	deletion present
GST positive genotype (+)	no deletion present
OR	odds ratio
CI	confidence interval
FDR	false discovery rate
